# Surface Plasmon Scattering in Exposed Core Optical Fiber for Enhanced Resolution Refractive Index Sensing

**DOI:** 10.3390/s151025090

**Published:** 2015-09-29

**Authors:** Elizaveta Klantsataya, Alexandre François, Heike Ebendorff-Heidepriem, Peter Hoffmann, Tanya M. Monro

**Affiliations:** 1 Institute for Photonics and Advanced Sensing, The University of Adelaide, Adelaide, SA 5005, Australia; E-Mails: alexandre.francois@adelaide.edu.au (A.F.); heike.ebendorff@adelaide.edu.au (H.E.-H.); peter.hoffmann@adelaide.edu.au (P.H.); tanya.monro@adelaide.edu.au (T.M.M.); 2 ARC Centre of Excellence for Nanoscale BioPhotonics (CNBP), The University of Adelaide, Adelaide, SA 5005, Australia; 3 Adelaide Proteomics Centre, The University of Adelaide, Adelaide, SA 5005, Australia; 4 University of South Australia, Adelaide, SA 5001, Australia

**Keywords:** microstructured optical fiber, surface plasmon resonance, surface plasmon scattering, plasmonics, biosensing

## Abstract

Refractometric sensors based on optical excitation of surface plasmons on the side of an optical fiber is an established sensing architecture that has enabled laboratory demonstrations of cost effective portable devices for biological and chemical applications. Here we report a Surface Plasmon Resonance (SPR) configuration realized in an Exposed Core Microstructured Optical Fiber (ECF) capable of optimizing both sensitivity and resolution. To the best of our knowledge, this is the first demonstration of fabrication of a rough metal coating suitable for spectral interrogation of scattered plasmonic wave using chemical electroless plating technique on a 10 μm diameter exposed core of the ECF. Performance of the sensor in terms of its refractive index sensitivity and full width at half maximum (FWHM) of SPR response is compared to that achieved with an unstructured bare core fiber with 140 μm core diameter. The experimental improvement in FWHM, and therefore the detection limit, is found to be a factor of two (75 nm for ECF in comparison to 150 nm for the large core fiber). Refractive index sensitivity of 1800 nm/RIU was achieved for both fibers in the sensing range of aqueous environment (1.33–1.37) suitable for biosensing applications.

## 1. Introduction

The widespread use of refractometric sensing devices based on SPR phenomenon can be attributed to its high sensitivity, immediate response, and simplicity of implementation [1]. Being a label-free transduction mechanism that determines changes of refractive index through a resonant wavelength shift, SPR allows for real time quantitative measurements that enable the detection of specific biomolecules for biomedical diagnostic applications [2,3] as well as the characterization of binding constants (association and dissociation constants) for biomolecular interactions studies [4].

Surface Plasmon Resonance (SPR) refers to the collective oscillation of free electrons at a metal-dielectric interface stimulated by incident light. For the resonant condition to occur, the propagation constant of the incident light has to match the propagation constant of the surface plasmons. This can be accomplished using a glass prism, and this approach is known as the Kretschmann SPR configuration [5]. While this method remains the gold standard for commercial SPR systems, they are bulky, expensive and not suitable for some applications such as remote sensing. To overcome these limitations, waveguides and optical fiber based SPR sensors have been developed, with the fiber/waveguide fulfilling the role of the prism for coupling the incident light with the plasmons [6]. Using optical fibers also enables development of alternative strategies for either exciting the plasmons or improving sensing performance. Some examples include bent fibers [7], tilted fiber Bragg grating SPR sensors [8], tapered probes [9,10], and many other more exotic architectures [11,12,13,14,15,16,17,18].

The sensitivity of refractive index sensors ($S_{\lambda }=\delta \lambda /\delta n$) is often used solely to characterize their performance. This ignores the sensor’s resolution, which is critical to the performance of the device. The resolution (*R*), defined as the smallest wavelength shift detectable by a sensing platform, depends on the optical setup and on the linewidth of the SPR signature. Resonance curves with narrower linewidths filter spectral noise more efficiently. This leads to lower spectral deviation from the actual position of an SPR peak making a very slight spectral shift to be resolvable [19,20]. Although a more comprehensive expression for the resolution of an SPR sensor can be found elsewhere [5,19], for the purposes of this paper it is sufficient to state that *R* of a sensor is directly proportional to the full width at half maximum (FWHM) of its SPR response. However, there is a trade off between sharper response curves and lower signal to noise ratio (SNR).

Sensitivity and resolution determine one critical performance characteristic—the detection limit (DL). The DL of an SPR sensor (DL$=R/S_{\lambda }$) is the smallest change of the refractive index that could be measured. Therefore, to improve the sensor’s DL, one would have to increase the sensitivity of the sensor, or reduce the resolution by either minimizing FWHM of SPR response or/and increasing SNR.

Sensitivity of an SPR sensor is highly dependent on the material of the fiber core and metal that supports surface plasmons. Higher sensitivity is generally attributed to lower refractive index fiber sensors. Using low refractive index glass results in shifting of the resonance towards longer wavelengths and an increase in the sensitivity but also causes broadening of SPR response leading to deterioration of the sensor resolution [21]. Silver is also known to provide better sensitivity in comparison to gold and other plasmonic metals due to its high SPR ratio (ratio between the absolute values of the real and imaginary parts of the dielectric constant) [22,23]. To overcome the trade-off between resolution and sensitivity, alternative fiber designs and sensing architectures have been explored. For example, reducing the fiber core diameter decreases the number of modes whose propagation constants match the propagation constant of surface plasmons. As a result, the linewidth of the SPR signal is reduced, enabling improved resolution in determination of the wavelength shift [24]. However, due to additional fiber processing requirements and the inherent fragility of nanowires or tapered fibers, using those sensors in a real application presents a significant challenge.

The SPR fiber sensor presented here relies on surface plasmon scattering for analyzing the SPR signal, the architecture previously described in [25,26,27]. The scattering is induced by high surface roughness of the silver coating deposited using an electroless plating method described in Section 2.2. While SPR scattering in optical fibers presents some advantages when compared with standard transmission measurements [25,26], using a rough metallic coating results in an increase of the SPR signal’s linewidth [28,29], which is detrimental to the overall sensor performance. This disadvantage can be alleviated by using a fiber with smaller core to enhance sensor resolution.

In this paper, we report an experimental demonstration of an SPR sensor based on silica Microstructured Optical Fiber with a core exposed along the fiber length (ECF). The relatively small core of 10 μm diameter is supported by an outer silica structure providing robustness to the fiber. The performance of the ECF SPR sensor is compared with a larger (∅ = 140 μm) multimode unstructured silica bare core fiber. As the presented fiber sensor architecture relies on surface plasmon scattering, further improvement in sensor resolution is provided by improved SNR. We also discuss other advantages and limitations of the scattering based SPR platform in relation to usage of small core fibers.

## 2. Experimental Section

### 2.1. Exposed Core Optical Fiber

**Figure 1:**
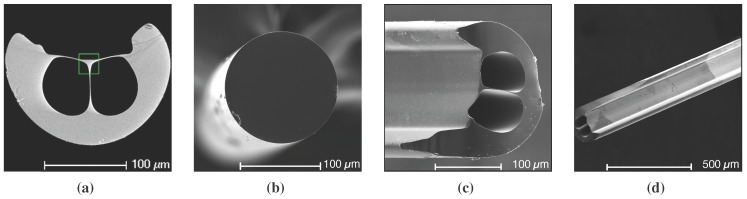
Scanning Electron Microscopy (SEM) images of optical fibers used for Surface Plasmon Resonance (SPR) sensor fabrication. (**a**) Exposed core fibre (ECF) cross section. Green rectangle shows the triangular core; (**b**) Bare core fiber cross section (shown for comparison); (**c**,**d**) Silver film fabricated on ECF (appears as lighter regions). The film fabricated around the outer fibre surface and the flat region where the core is located.

To analyze the influence of the fiber core diameter on SPR sensor performance, two different fibers, both made in-house out of F300 silica preform (Heraeus Quarzglas GmbH & Co.KG, RI of 1.457 at 633 nm, Kleinostheim, Germany), have been used to fabricate the sensors. One fiber is an ECF with a 10 μm core supported by thin struts within a 200 μm support structure [30]. The ECF is a modified suspended core fibre with an asymmetric geometry where one side of the core is exposed along the whole fibre length as shown in Figure 1a. Due to the specific ECF geometry, no additional fibre processing, such as side polishing or tapering, is required to access the core to allow for metal deposition. Another fiber is a bare core fiber with a core diameter of 140 μm as shown in Figure 1b for comparison.

The estimated number of modes in a silica fiber of 10 μm core diameter and air cladding is 670, compared to the 140 μm bare core fiber with cladding RI of 1.375, which supports over 42,000 modes. The calculation of the number of modes is performed for a wavelength of 650 nm using numerical simulation for an unstructured air-clad fiber. Even though the simulation assumes a circular fiber core while the ECF core is analogous to a triangular cross section of suspended core fibers [31], this simple calculation is sufficient for estimating the number of modes that could be supported by ECFs.

### 2.2. Sensor Fabrication

A thin silver film of approximately 50 nm thickness was deposited on a 1 cm-long section around each fiber sample using an electroless plating method. As the ECF’s core is exposed to an external environment, the sensor fabrication procedure is essentially the same for both, bare core and ECF sensors. A silver film fabricated on ECF is shown in Figure 1c,d.

First, the fiber samples were thoroughly cleaned with methanol and deionized water followed by washing in a detergent (Decon90), rinsing in deionized water, and finally dried. After the cleaning process, the fiber surfaces were sensitized to promote silver film adhesion with 0.1 M of stannous chloride solution ($SnCl_{2}$) in 0.1 M aqueous solution of hydrochloric acid (HCl). The fiber samples were soaked in the sensitizer for 5 min then rinsed with deionized water. The mechanism behind the sensitization process involves precipitation of soluble tin ions from the liquid layer adhering to the dielectric substrate during water rinsing due to rapid pH increase [32]. This pre-treatment step is required to chemically modify the silica surface prior electroless plating of silver on the silica substrate. It is speculated that tin ions absorbed on a glass surface act as nucleation sites onto which silver may be deposited [33].

The electroless plating process, also known as Tollens silver mirror reaction, is described in [34] and consists of the deposition of a thin film via precipitation of metallic silver from ammoniacal silver nitrate solution in the presence of a reducing agent. Tollens reaction parameters were adjusted to produce silver coatings of around 50 nm on fibres and glass slides. The coating thickness was characterized using Atomic Force Microscopy (AFM). In addition, the thickness characterization on glass slides was performed through transmission measurements using HeNe laser (λ = 633 nm). Calculation of the thickness of the films was carried out using Beer-Lambert law: $I=I_{0}e^{-t\alpha }$, where *I* and $I_{0}$ are incident and transmitted power respectively, *t*—film thickness, and α = 756,310 cm${}^{-1}$—absorption coefficient of silver at 633 nm [35]. The results for the silver film thickness acquired with the transmission measurements agree with those obtained using microscopy and, therefore, further thickness control of every sensor batch was estimated by measuring thickness of the silver coatings on glass slides prepared together with the fiber samples.

Even though the ECF has a small core held up by thin struts, the support structure of 200 μm diameter provides sufficient mechanical strength. There is no specific handling required to produce SPR sensors using ECF and the silver coating and fibre preparation procedures are similar to those used with large bare core sensors. The fabrication yield of the ECF SPR sensors is comparable to bare core SPR sensors: up to 10–15 fibres can be simultaneously coated in one chemical deposition reaction regardless of the fibre type.

### 2.3. Experimental Setup

**Figure 2:**
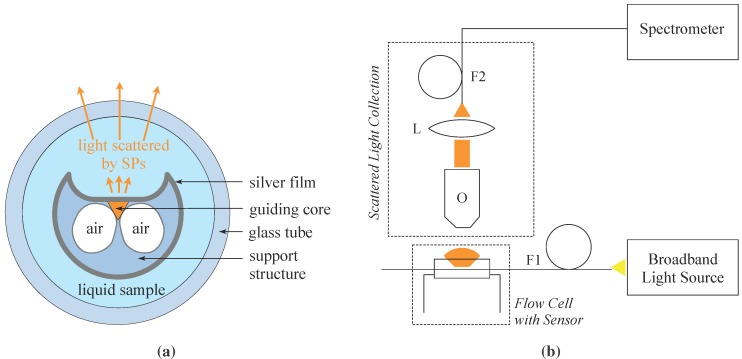
(**a**) Cross section of a flow cell with enclosed ECF SPR sensor (not to scale) where Surface Plasmons (SPs) are excited on the exposed part of the fibre core; and (**b**) Optical setup for SPR sensors characterization. F1—optical fiber with a sensing region enclosed in a flow cell, F2—optical fiber Ocean Optics with 200 μm core diameter, L—converging lens, O—microscope objective ×20, Numerical Aperture 0.4. Broadband light used to excited SPR is shown in yellow. Light scattered by the plasmons is shown in orange.

To perform SPR characterization of the prepared fiber probe, the sensing region was enclosed in a flow cell that was constructed from a glass capillary tube allowing to flow liquid samples along the exposed core of the fiber sensor. Having one side of the ECF core exposed to the environment allows to perform sensing without filling the air holes which significantly simplifies the sensing apparatus. A schematic of a cross section of the flow cell with enclosed ECF SPR sensor is presented in Figure 2a. Plastic tubings were inserted for the liquid inlet and an outlet at two opposite ends of the tube. The flow cell was sealed using a low refractive index curable adhesive (MY-133-V2000, My Polymers Ltd., Nes Ziona, Israel). Serial dilutions of glycerol in deionized water (from 0% to 30%) with refractive indices ranging from 1.330 to 1.372 were used as liquid dielectric samples for RI measurements. The small range of the refractive indices close to aqueous environment used in the experiments was chosen to simulate refractive index changes that often occur in biosensing applications.

The setup used for SPR sensor characterization is schematically shown in Figure 2b. A supercontinuum light source (KOHERAS SuperK Compact, NKT Photonics A/S, Birkerød, Danmark) was coupled into the fiber (F1) with the sensing region enclosed in the flow cell. The scattered light collection apparatus consisted of an objective (O), a converging lens (L), and an optical patch cord cable (F2) to transfer the collected light to an Ocean Optics compact spectrometer (QE 65000) for analysis. Light transmitted through the optical fiber was projected onto a screen to ensure coupling into the core as opposed to the support structure in the case of ECF based sensors.

### 2.4. Data Analysis

Scattered spectra were recorded for every sample’s RI and for intermediate water rinsing steps to ensure thorough removal of a previous sample. The spectral position of a peak in the scattered signal was found by fitting collected data with a Gaussian function. The wavelength shift with respect to the change in the RI of the sample surrounding the sensing region was plotted and the slope of the linear fit of the curve was considered to be the sensor’s sensitivity value. FWHM was found from the Gaussian fit for each peak as $2\sqrt{-2\ln(0.5)}\sigma$, σ being square root of the variance, and the mean of values for every sample was taken as a FWHM for a sensor. Full width at ninetieth of the maxima (FWNM or $\Delta \lambda_{0.9}$) was evaluated as $2\sqrt{-2\ln(0.9)}\sigma$. To consider both performance identifying parameters, a Figure of Merit (FOM) was defined as the ratio between the sensitivity of a sensor and the average FWHM of SPR response curves (FOM$=S_{\lambda }/\Delta \lambda_{0.5}$). Height of a peak was measured as maximum intensity in arbitrary units (a.u.) keeping integration time of the spectrometer constant. SNR for each sensor was calculated as the mean of the squared signal amplitude divided by the mean of the squared noise amplitude for each data point averaged over all SPR peaks for each RI.

Errors in resonant wavelength shift and FWHM were calculated by combining experimental uncertainties and fitting errors. Water rinsing steps were used to estimate experimental errors. In particular, the sum of deviations of the position of a peak at rising steps before and after each glycerol concentration from the original step for water was considered an experimental uncertainty. Those errors mainly come from a change in coupling conditions due to a stage drift or a fiber movement, and a change in the alignment of the sensing region under the collection setup during the experiment. Errors in refractive indices of the prepared glycerol solutions were calculated as a sum of uncertainties in the solution RI due to thermal fluctuations, spectral dispersion, and preparation of the serial dilutions of glycerol.

To estimate fitting errors of the experimental data, a threshold parameter, that is a fraction from the maximum height of a peak where data for the fit has been taken from, was used. Errors in the fit for a peak wavelength and FWHM were estimated as scatter of the values (standard deviations) of the three different fits: broad fit that considers larger number of points around the maxima, narrow fit with less number of points, and a fit between those two at which all of the parameters ($S_{\lambda }$, FWHM, FWNM, Height, and FOM) were evaluated.

## 3. Results and Discussion

**Figure 3:**
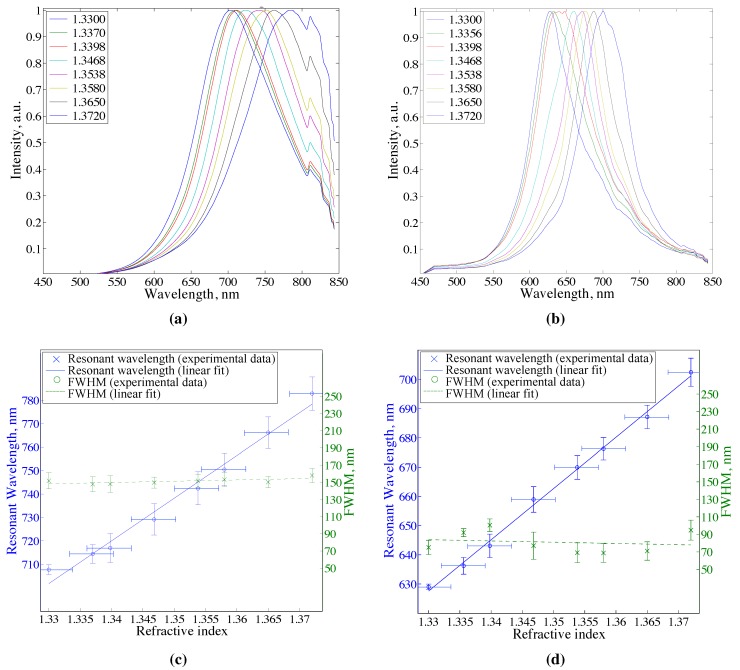
Normalized scattered spectra for (**a**) bare core fiber SPR sensor and (**b**) exposed core fibre (ECF) SPR sensor. Legends show the values of glycerol solutions’ refractive indices used in the experiment. Sensitivity curves and full width at half maximum (FWHM) for (**c**) bare core SPR sensor and (**d**) ECF SPR sensor. Bare core sensor: core diameter 140 μm, silver film thickness 57 nm. ECF sensor: core diameter 10 μm, silver film thickness 55 nm.

Bare core fiber SPR sensors of 140 μm core diameter were compared to sensors fabricated using a ECF of 10 μm core prepared following the same procedure. Experimentally obtained spectra for the bare core fiber sensor exposed to the different refractive index solutions are presented in Figure 3a. Resonant wavelength with respect to the refractive index of the sample (sensitivity curve) and FWHM of the scattered signal are shown in Figure 3c. The slope of the sensitivity curve yields the sensitivity of 1823 ± 58 nm/RIU while the mean value of the FWHM is calculated to be 149 ± 8 nm for the sensor. The small secondary peak at around 820 nm in the Figure 3a is scattered light from the light source pump and is not related to the SPR signal. The results of the experiments that were conducted using an ECF are presented in Figure 3b,d. Sensitivity of the ECF SPR sensor is 1753 ± 27 nm/RIU and the mean value of the FWHM is 75 ± 10 nm. Spectra presented in Figure 3a,b are scattered light intensities normalized to their maxima with moving average function applied to smooth data fluctuations. The values for sensitivity, FWHM, and other performance parameters are evaluated from Gaussian fits as described in Section 2.4.

Table 1 summarizes the performance characteristics of the two types of sensors, bare core SPR sensor and ECF SPR sensor. The sensitivities of both devices are the same within their uncertainty ranges. As far as the linewidth is concerned, ECF SPR sensor manifests twice as narrower scattered peaks than the bare core SPR sensor which is expressed in the variable FWHM and FWNM. This makes smaller wavelength shifts of the plasmonic resonance resolvable leading to an improvement in ECF sensor resolution and detection limit, and an increase in FOM by at least a factor of two. For the comparative analysis multiple ECF and bare core SPR sensors have been prepared and characterized and the performance parameters have shown sufficient reproducibility.

**Table 1:** SPR performance characteristics of the bare core fibre sensor and the exposed core fibre (ECF) sensor.

Performance/Parameters	Bare Core Sensor	ECF Sensor
Core diameter (∅), μm	140	10
Silver film thickness (*t*), nm	57	55
Peak wavelength ($\lambda_{res}$), nm for RI 1.33	708 ± 2	629 ± 1
Sensitivity ($S_{\lambda }$), nm/RIU	1823 ± 58	1753 ± 27
FWHM ($\Delta \lambda_{0.5}$), nm	149 ± 8	75 ± 10
FWNM ($\Delta \lambda_{0.9}$), nm	58	29
Height, a.u.	51,539	997
SNR, dB	33	27
FOM, RIU${}^{-1}$	12 ± 1	23 ± 3

The experimentally demonstrated results for the ECF SPR sensor presented here are, in general, in agreement with previously reported theoretical work based on numerical simulation of SPR in ECFs [36,37]. The predicted sensitivity of a silver coated SPR sensor based on an ECF of similar geometry is 2000–4000 nm/RIU for the range of samples’ RIs between 1.33–1.37, and the FWHM of SPR response is around 50 nm [37]. Despite the slightly lower performance of our experimentally demonstrated ECF sensor, which is likely to be attributed to a different dielectric function of the silver coating produced by chemical electroless plating and the roughness of the film, the experimental values obtained for the ECF SPR sensor support the results previously predicted by the modelling.

Further analysis of experimental data suggests that a drawback of using the small core ECF sensor is its lower SNR of 460 in comparison to SNR of 2050 estimated for the bare core fibre sensor. An increased noise causes an increased uncertainty in determining an extremum in the spectral response leading to a deterioration of the sensor resolution. Therefore, depending on the contribution of the reduced SNR towards resolution, narrower linewidth of the SPR response might or might not result in an improvement of the sensor resolution. Considering the numerically simulated expression presented in [19] ($R=3\sigma \approx 3(\Delta \lambda /\left(4.5\;S\;N\;R^{1/4}\right))$, where Δλ is FWNM), we estimated the resolution for the bare core and ECF sensors as 5.75 nm and 4.17 nm respectively. This would give the detection limit of 0.0032 RIU for the bare core sensor and 0.0023 RIU for the ECF leading to the improvement factor of 1.4 for the ECF sensor. On the other hand, the experimental contribution of the SNR might differ from the numerically predicted dependency. Specifically, curve fitting for data analysis is influenced by both parameters, linewidth and SNR. The average fitting error in determining the spectral position of the peaks for the bare core sensor was estimated to be 2.47 nm, while this for the ECF sensor is only 1.21 nm which approaches the spectral resolution of the spectrometer. While the calculated values for the resolution and the detection limit are somewhat speculative, the analysis shows the trend that the reduced linewidth of smaller core SPR sensors tend to improve the resolution and the detection limit, even in the presence of increased noise level. The latter could be potentially alleviated by the choice of light source used to excite SPR.

Based on the results of the described experiments one may conclude that the structural parameters of the fiber have a strong influence on the sensor performance as well as on the scattered peak position. The observed wavelength is the result of the convolution of different resonance peaks each caused by a specific resonance condition satisfied by a given mode-wavelength combination. The number of modes contributing to the resonance could be one of the potential reasons for broadening of SPR peaks. It has been noticed that in a highly multimode fiber with thousands of modes, such as the bare core fiber used in the experiments, the coupling conditions do not have a strong effect on the observed SPR wavelength. However, for the smaller core multimode fiber, such as the ECF used here, we registered a stronger dependency of the scattered peak wavelength on the properties of the guided light, which in turn is influenced by coupling. Thus, in order to achieve coupling consistency between different fiber sensors, we aimed at maximizing intensity of the signal scattered from the sensing region.

**Figure 4:**
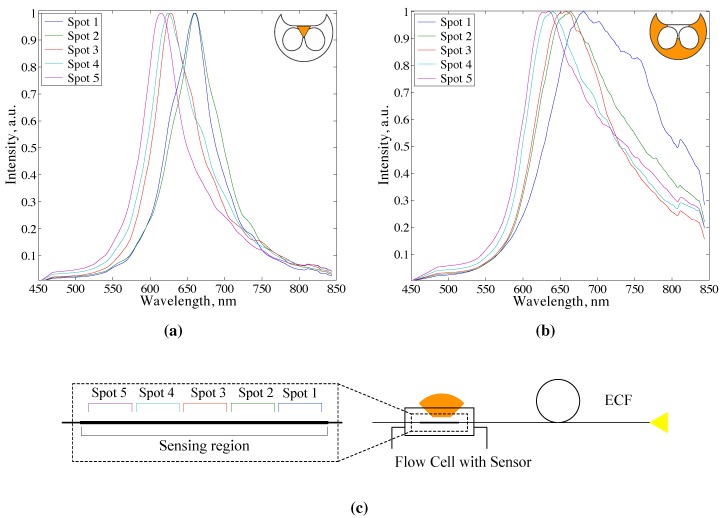
Scattered spectra taken from different spots on a sensing region of the exposed core fibre (ECF) SPR sensor in case of (**a**) core coupling, and (**b**) support structure coupling (as shown on the insets). Refractive index of the outer dielectric medium is 1.33; (**c**) Schematic diagram of five equidistant spots on the sensing region of ECF SPR sensor. Sensing region length is 15 mm.

In addition to the coupling efficiency, properties of the guided light are affected by interaction with the metal coating itself. SPR is a lossy process that causes attenuation of electromagnetic radiation near the resonance frequency. After the guided light undergoes an interaction with the metal film, its spectral properties change. As a result, the observed wavelength of the peak in the scattered signal from a particular spot on the sensing region shifts depending on the losses that occurred in the fiber prior to that spot. This concept is demonstrated by comparing spectra taken from five equidistant positions along a sensing region. Figure 4a presents normalized scattered spectra for ECF SPR sensor collected from different spots on the sensing region of 15 mm length with the same outer dielectric medium. As longer wavelengths are scattered at the beginning of the sensing region, the peak shifts towards the shorter wavelengths. This is also noticeable when light is launched into the support structure (the cladding of the microstructured fiber) rather than the small core (Figure 4b). The 200 μm diameter support structure is sufficiently large that it could be considered as a highly multimode fiber. Sensor spot positions from which the spectra were taken from are depicted in Figure 4c. This effect of shifting the resonant wavelength with a position on the sensor is an artifact of the scattered interrogation method and could be mitigated by collecting signal from a larger area of the sensor or reducing the length of the sensing region. Although we verified that the sensor sensitivity for both coupling conditions does not strongly depend on the position on the sensing region and the observable peak wavelength, the linewidth of the scattered peaks might be affected. In the case of the larger core sensor (support structure coupling), FWHM is larger at the first spot on the sensing region reaching around 180 nm, then reducing down to 150 nm at the further spots (Figure 4b). The broadening of the peak at the beginning of the sensing region for larger core SPR sensors might be attributed to the larger scattering area that introduces more scattered background unrelated to SPR at the boundary of the coated region. This effect is not noticeable for the small core sensor (core coupling condition) where FWHM does not vary with the position on the sensing region (Figure 4a). In practice, this may signify the importance of ECF SPR sensors for usage in multiplexed sensing architectures where spatially localized specific antibodies along one long sensing region or multiple sensing regions are used for detection.

## 4. Conclusions

In this work, we have experimentally demonstrated an SPR sensor with a reduced linewidth that is fabricated using ECF with a small core. The refractive index sensitivity of the ECF SPR sensor is 1800 nm/RIU, comparable with the sensitivity of a large bare core fiber SPR sensor. The ECF sensor exhibits narrower SPR curves with FWHM of 75 nm, a factor of two improvement in comparison to the previously reported large core SPR sensor. Even though the performance of the reported ECF sensor is comparable to the current state of the art fibre optic SPR sensors [38], there is a significant scope for performance improvement, such as further reduction in linewidth, increased durability, enhanced sensitivity, as well as a more sophisticated collection apparatus. A further reduction in the linewidth could potentially be achieved by using even smaller core ECF with a core diameter down to a couple of micron which would support just a few fiber modes. The use of a microstructured fiber allows for easier sensor fabrication and more practical applications in comparison to fragile free standing nanowires or tapered fibers. The architecture utilizes spectral interrogation of the scattered plasmonic wave mediated by rough metallic coating which provides additional practical advantages such as reduced SNR, lower dependency of the sensor performance on the coating thickness, and possibility of combining SPR with other phenomena, such as fluorescence, in a single device. The proposed SPR configuration is promising for applications such as biological or chemical sensing where improved resolution is required.
